# PReS-FINAL-2153: Intestinal gamma/delta- T cells are upregulated in children with active uveitis

**DOI:** 10.1186/1546-0096-11-S2-P165

**Published:** 2013-12-05

**Authors:** M Arvonen, P Vähäsalo, T Karttunen

**Affiliations:** 1 Pediatrics, Kuopio University Hospital, Kuopio, Finland; 2 Pathology, University of Oulu, Oulu, Finland; 3 Pediatrics, Oulu university hospital, Oulu, Finland

## Introduction

In experimental uveitis, activated γ/δ- T lymphocytes produce cytokines, which contribute to differentiation of pathogenic effector T cells. In addition mucosal tolerance in experimental uveitis can be induced by introducing uveitogenic retinal soluble antigen orally and this phenomenon is dependent on γ/δ T cells. The major reservoir for γ/δ- T cells is intestinal mucosa, where they function in safeguarding the integrity of the epithelial barrier. Abnormal activation of these intraepithelial γ/δ- T-cells may contribute to immunopathological responses which can even present themselves as extraintestinal manifestations. ANA-positive in JIA is associated both with uveitis and accumulation of γ/δ- T cells with gut-associated subtype in synovium.

## Objectives

Our objective was to assess the potential association of intestinal mucosal T-cell subsets with uveitis in patients with JIA and gastrointestinal symptoms.

## Methods

Duodenal mucosal samples were taken from patients with JIA and gastrointestinal symptoms (n = 13). Α/β- T cells and γ/δ- T cells were immunohistochemically stained in frozen sections. Intestinal mucosal γ/δ- and α/β- intraepithelial cells were quantitated from duodenal mucosal samples by an assessor blinded for all clinical data.

## Results

Patients with active uveitis (n = 4) during endoscopy had higher α/β- T cell (19, 14-23) and γ/δ- T cell counts (2.7, 2.1-3.4) in duodenal biopsies compared with patients without uveitis (n = 9) (13, 4-22; p = 0.05; 0.7, 0- 4.9; p = 0.034; Mann-Whitney U-test).

## Conclusion

Our finding suggests, that intestinal immune activation involving the increase of γ/δ lymphocytes contributes to the pathogenesis of uveitis in children with JIA. Although both the factors inducing the intestinal mucosal immune activation in JIA and the mechanism linking intestinal activation and uveitis remain speculative, we suspect that pathological entry of the gut originating lymphocytes into the iris is possible because intensive distribution of the adhesion molecules on the endothelium of the vessels in the iris in patients with uveitis. Our finding encourages to study the potential role of gastrointestinal factors in the pathogenesis of uveitis associated with JIA. Our finding together with reports of experimental autoimmune uveitis models support the hypothesis of gut mucosa, gut lymphocyte homing receptors in the uvea, and the role of gut originating lymphocytes in the development of uveitis.

## Disclosure of interest

None declared.

